# Experimental Study of the Effect of Internal Defects on Stress Waves during Automated Fiber Placement

**DOI:** 10.3390/polym10040413

**Published:** 2018-04-09

**Authors:** Zhenyu Han, Shouzheng Sun, Wenqi Li, Yaoxu Zhao, Zhongxi Shao

**Affiliations:** 1 School of Mechatronics Engineering, Harbin Institute of Technology, No. 92, Xidazhi Street, Harbin 150001, China; hanzy@hit.edu.cn (Z.H.); sunsz@stu.hit.edu.cn (S.S.); zhaoyaoxu@163.com (Y.Z.); 2 SAIC Motor Passenger Vehicle Company, No. 201, An Yan Road, Shanghai 201804, China; wenqi268@126.com

**Keywords:** carbon fiber reinforced thermoset composites, automated fiber placement (AFP), stress wave, defects

## Abstract

The detection technique of component defects is currently only realized to detect offline defects and online surface defects during automated fiber placement (AFP). The characteristics of stress waves can be effectively applied to identify and detect internal defects in material structure. However, the correlation mechanism between stress waves and internal defects remains unclear during the AFP process. This paper proposes a novel experimental method to test stress waves, where continuous loading induced by process itself is used as an excitation source without other external excitation. Twenty-seven groups of thermosetting prepreg laminates under different processing parameters are manufactured to obtain different void content. In order to quantitatively estimate the void content in the prepreg structure, the relation model between the void content and ultrasonic attenuation coefficient is revealed using an A-scan ultrasonic flaw detector and photographic methods by optical microscope. Furthermore, the high-frequency noises of stress waves are removed using Haar wavelet transform. The peaks, the Manhattan distance and mean stress during the laying process are analyzed and evaluated. Partial conclusions in this paper could provide theoretical support for online real-time detection of internal defects based on stress wave characteristics.

## 1. Introduction

**Figure 1:**
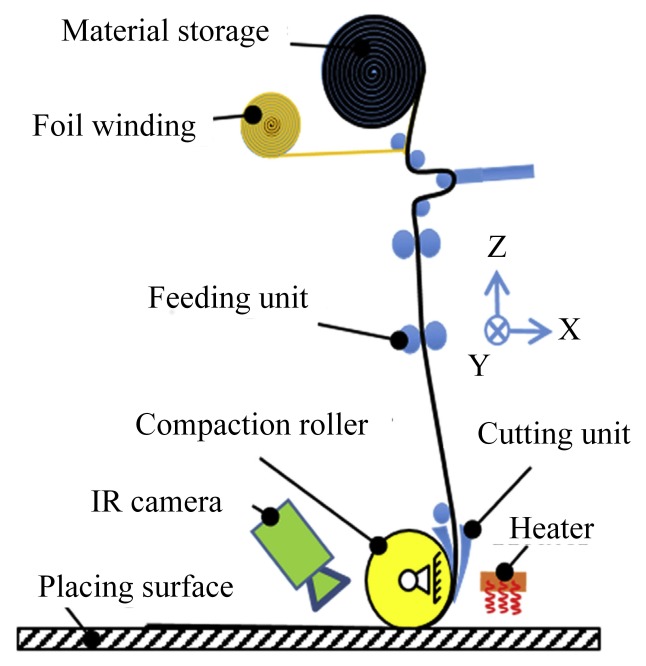
The principle of AFP process.

Polymer composites have been widely used in aerospace, naval, automotive, and civil applications [1,2,3] due to their high stiffness to weight ratio, high strength to weight ratio, greater fatigue resistance, better long-term stability, lower thermal expansion and excellent corrosion resistance compared with metal-based traditional materials [4,5]. The composite manufacturing process refers to using a certain method to laminate and consolidate the prepregs for further improving mechanical properties. This process can be conducted by using a variety of manufacturing methods, such as filament winding, tape/fiber placement, and resin transfer molding. Among these methods, automated fiber placement (AFP) [6,7,8] is an advanced composite manufacturing technology. An AFP machine consists of a placement head, and auxiliary mechanical and electrical structures to lay prepreg tows onto a mold to construct the prepreg laminates. For each ply, the machine accurately places the tows on the mold with the proper angles. The part is then placed in an autoclave to realize the cross-linking reaction of polymer materials for curing the plies [9,10,11]. AFP is suitable for parts that vary greatly in aspect of appearance and size [12,13,14]. Their productivity is 5–20 times higher than manual fiber placement or semi-AFP [15]. The automated fiber placement process [16] is shown in Figure 1.

Manufacturing defects are driven by complex loads [17,18], placing accuracy [19] and temperature profile during the AFP process, which result in the reduction of mechanical properties of the laminates, and even complete failure. The formation of defects is commonly divided into two stages for thermosetting materials. In the first stage, preheat softening, compression deformation, and matrix flow of prepregs in the laying process could generate the initial defects. The second stage refers to the formation of final defects caused by the heating, pressurization and cooling in the curing process. The redistribution of the stress in the laminated component or the cross-linking chemical reaction of the matrix could result in the formation of the final defects. This paper focuses on the relationship between the initial defects in the first stage and stress waves. Internal defects in this paper include pore defects, shrinkage cavities, and crack defects. Stress wave propagation in the materials can not only reflect the characteristics of their structure, but also interact with the media to further affect their internal structure. Some publications show that the formation and evolution of defects are caused by the coupling of multiple stresses and their dynamic waves during the manufacturing process. For example, microcrack initiation and its expansion are due to the difference of the thermal expansion coefficient between the fibers and the resin. In the matrix exists some tensile stress during the laying process, which results in the initial cracks in the matrix and local cracks in the interface of fibers and matrix [20]. In addition, the defects can further change the characteristics of stress wave propagation. Taking advantage of this feature, the stress waves are widely used in non-destructive testing in the field of geological exploration [21,22,23], tree damage diagnosis [24,25,26], and defect detection of composites [27].

Wang and Chang [28] used the high-order plate theory to model the wave signal scattered by the delamination damage. In their study, Kane-Mindlin theory was extended to transversely-isotropic materials to study the scattering of typical waves in the plane. Tasdemirci and Hall [29,30,31,32] published a series of papers on the characteristics and distribution of stress wave propagation in lightweight armored laminates. The stress distribution characteristics of layered composite structures composed of metal, ceramic, and ethylene propylene diene monomer (EPDM) rubber under one-dimensional stress were discussed by using the Split Hopkinson Pressure Bar (SHPB) experiment and numerical simulation. The effect of low-impedance medium, energy-absorbing medium on stress wave were analyzed. Gama [33] used the manganese-copper probe to measure the stress wave propagation in the layered composite structure composed of high-strength armor steel, alumina ceramic, closed-cell foam aluminum, and aluminum alloy under the impact of the missile. He pointed out that open-cell foam aluminum plays a decisive role in the attenuation of stress waves in composite structures. Some publications [34,35,36,37] have studied the damage detection and identification of the cured composite materials based on the stress wave (Lamb wave) and ultrasonic characteristics by means of external excitation. Some results show that the wave can be scattered around the defect or damage, resulting in energy wave attenuation. Specifically, in the same material and propagation distance, the characteristics of energy waves are weakened in the testing point, including strain energy, mean stress, wave peak, wave velocity, etc. However, due to the coupling of multiple processing parameters during the laying process, the stress wave signal is complicated and the characteristics of the waves are not obvious. Additionally, the online quantitative identification of the initial defect is difficult to determine. Therefore, the relationship between the initial defect and the stress wave during the laying process is rarely studied. Whether it is consistent with the regularities of waves tested in the quality control of the cured composites remains unknown. The detection study of defects in the laying process is currently deepening in the field of offline defects and online surface defects [13,38]. If the relationship between the stress wave and the internal defects in the laying process is revealed, it is possible to realize the real-time online detection and process control of the internal initial defects based on the stress wave characteristics.

The aim of this paper is to investigate the relationship between internal defects and stress wave propagation characteristics during the AFP process experimentally. The rest of this paper is organized as follows: In Section 2, Twenty-seven groups of AFP experiments are designed by determining different laying parameters. In Section 3, the real-time stress wave testing experiments during the laying processes are carried out. Prepreg laminates are then cured. According to an A-scan ultrasonic flaw detector and photographic methods by optical microscope, the relationship between the void content and ultrasonic attenuation coefficient is established to estimate the initial void content before curing. Lastly, the relationship between the initial void content and stress wave propagation characteristics, mainly peaks, Manhattan distance, and mean stress during the laying process, are analyzed and discussed.

## 2. Experimental Method

### 2.1. Parameters and Equipment

The 6511 type carbon fiber/epoxy prepreg, produced by Weihai Guangwei Composite Material Co., Ltd. (Weihai, China), is selected as the experimental material. Some typical properties of the test prepreg material are listed in Table 1.

In order to manufacture different void content of samples, three levels of testing are respectively cross-linked to obtain 27 groups of prepreg laminated samples in terms of three processing parameters, including laying speed (*S*), compaction force (*F*), and pre-heating temperature (*T*). The experimental parameters are shown in Table 2.

In Table 2, compaction forces ranged from 350 to 850 N in parentheses refer to the pressure in rolling cylinder. It is not the actual compaction forces between the roller and prepreg laminates. So a pressure sensor is attached on the laminates. Different cylinder pressures are then used to compress the pressure sensor to obtain the maximum pressure values between the roll and laminates, i.e., 0.5, 1.1, and 1.7 MPa. Considering safety, stability of the experimental platform and size effect, the pulse frequency of the UMAC control device is controlled, which can adjust the laying speed ranged from 0 to 20 mm/s.

**Figure 2:**
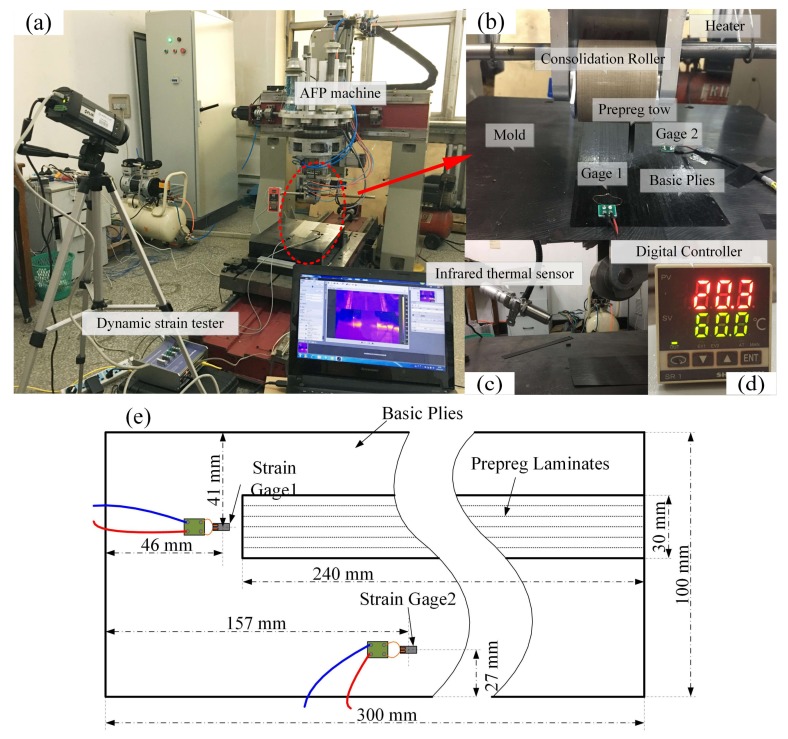
Twenty-seven groups of samples and curing equipment: (**a**) samples; (**b**) autoclave; (**c**) thermal sensor; (**d**) digital controller; and (**e**) sensor position.

A gantry-type five-axis automated fiber placement machine of independent development is capable of automatic shearing, clamping, and re-feeding for the prepreg tows, which is applied in the experiments. It integrates the pressure roller (*r* = 30 mm, rigid material), the preheating system, the laying system, etc. The surface of mold used by cast iron material is processed as horizontal and smooth. ASMD5A type multi-channel high-precision dynamic stress acquisition system, produced by SIGMA Co., Ltd. (Jinan, China), is used to collect the stress wave signal. The system can measure a variety of strain and stress state in solid materials. The sampling range can be adjusted from 5 Hz to 128 kHz. The sensor can use the strain gage (3 mm × 10 mm) or strain flower to measure stress states changed in many directions. Additionally, the strain resolution can reach 0.1 με. In order to compensate for the temperature drift during the experiment, half-bridge common compensation is used. In other words, two strain gages of the collected signal are compensated to reduce the amount of drift resulting from the temperature through a temperature compensation sensor. The laying speed and compaction force can be set up by the CNC system in the AFP machine. The precise control of the pre-heating temperature needs a temperature control system that forms a temperature feedback and control loop through an infrared probe (the range of 0–300 °C), relay, air switch, and heater. The experimental AFP machine and online testing equipment are shown in Figure 2.

### 2.2. Design

**Figure 3:**
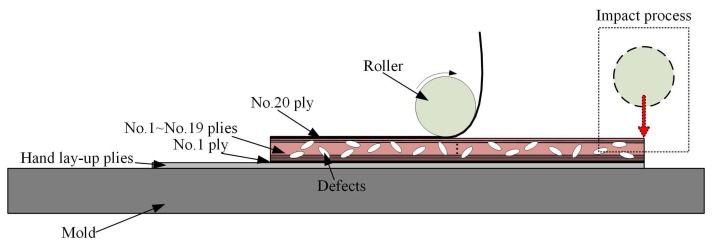
The experimental scheme.

**Figure 4:**
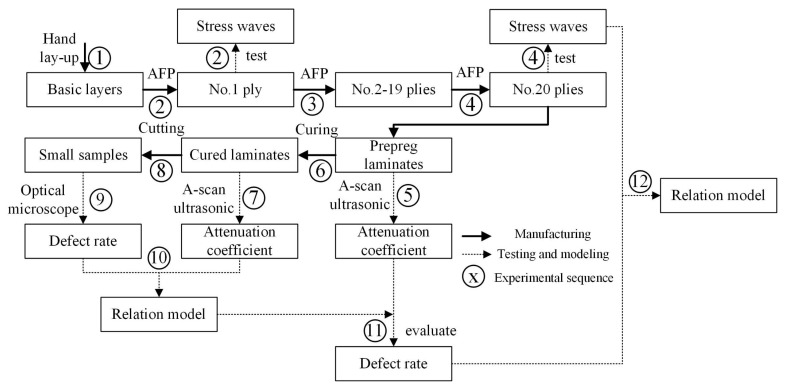
Experimental process and sequence.

The experimental scheme and purposes are introduced as follows:

(1) In order to avoid the errors between the mold and the prepreg because of the difference in elastic modulus, two basic prepreg layers (300 mm × 100 mm) are laid on the surface of the mold before the experiment for better avoiding the stress wave reflected from the mold. Two strain gages are attached to the surface of basic layers. Their positions are shown in the upper right of the Figure 2. This paper focuses on the characteristics of the stress shear wave (S wave) in the fiber direction.
(2) One single tow is placed in each laying process. Firstly, six tows with each width of 5 mm are laid in an orderly fashion under different processing parameters to form one layer with a total width of 30 mm, total length of 260 mm. The state of the stress waves during the laying process is examined. The relationship between processing parameters and characteristics of stress waves is explored. This paper does not discuss this relationship.
(3) No. 2–No. 19 plies are then laid to increase the thickness of samples about 2 mm under different processing parameters. Internal defects start to form and continue to accumulate during this process, in which we could obtain samples with different void content.
(4) The same processing parameters (laying speed of 20 mm/s, compaction force of 1.7 MPa, pre-heating temperature of 40 °C) are used to lay a tow on the surface of laminated prepregs with the sampling frequency of 8 kHz. This process can be considered to have a limited impact on the internal defects. Thus, the relationship between stress waves and internal defects can be further studied during the AFP process. The experimental scheme and process is shown in Figure 3 and Figure 4, respectively.

## 3. Curing and Defect Detection

This section is aimed at experimental steps of 6–11 in Figure 4. It is necessary for the curing process after the laying process, so that the components can be obtained as stable and high mechanical properties through cross-linking reactions by the internal polymer chains. However, the defects produced in the laying process are not the final defects of a component due to the implementation of the curing process. Therefore, it is impossible to investigate the relationship between the void content in the first stage and the stress waves during the laying process, which blurs the relationship between the stress waves and defects. In order to solve this problem, the relationship between the defects before and after curing need to be found. Defects before curing are further predicted using the defects after curing. In this section, ultrasonic testing is executed before and after curing. The relation model between the void content and the ultrasonic attenuation coefficient is quantitatively established combining ultrasonic testing after curing and off-line detection, which is applied to estimate the void content before curing.

**Figure 5:**
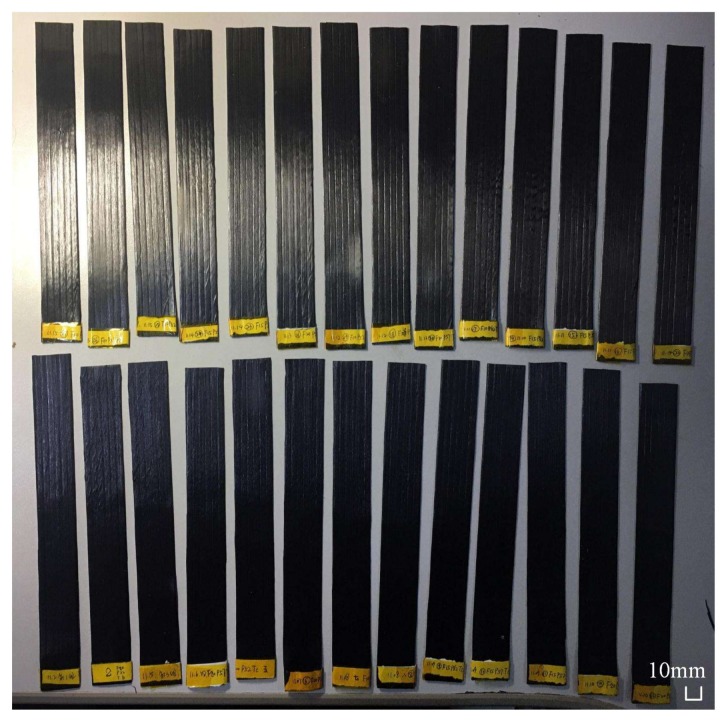
Twenty-seven groups of samples made by AFP.

To this end, the ultrasonic attenuation coefficients of the samples before and after curing are compared using the A-scan ultrasonic testing method, which is applied to analyze the relationship between the defects before and after curing. GE’s USD15 single-channel ultrasonic detection system (General Electric Company, Boston, MA, USA) is used as the ultrasonic A-scan equipment. The vacuum bag-autoclave curing method is used. Breather cloth is a kind of woven polyester fabric that is used during vacuum infusion to allow for the even removal of air from a vacuum bagged composite. Additionally, the same curing processing parameters, involving a curing pressure of 0.5 MPa, a vacuum pressure of 0.1 MPa, a curing temperature of 120 °C, and a curing time of 150 min, are set to cure Twenty-seven groups of samples by autoclave. The Twenty-seven groups of samples before curing are shown in Figure 5.

**Figure 6:**
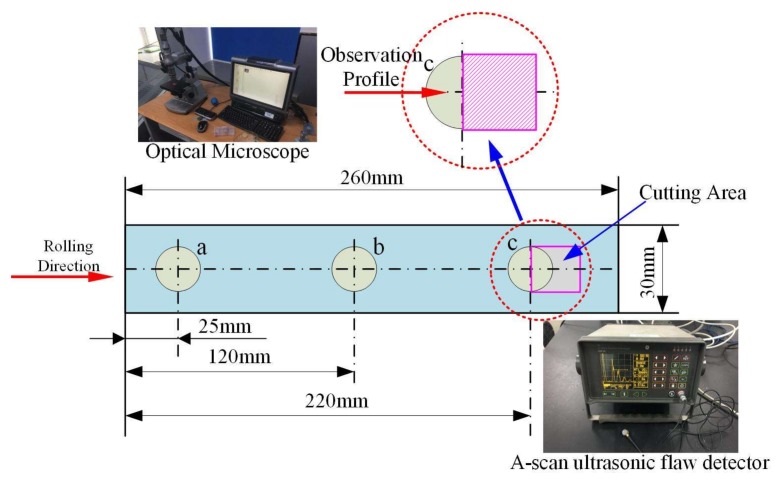
Offline detection method.

In Figure 6, we use an A-scan ultrasonic testing system to measure the attenuation rate of three testing points with a, b, and c before and after curing. After curing, the photographic method by optical microscope is used to quantitatively detect the void content of point c. The mathematical relation between void content and attenuation coefficient is then obtained. Furthermore, according to the attenuation coefficient before curing, the void content of a, b, and c before curing is estimated where the average value is used to evaluate the void content of the whole samples before curing. The specific implementation is as follows: the photographic method by optical microscope has one of the higher accuracies of void content offline defect detection, which can detect a void content value of less than 0.5%. The method is used as an experimental calibration method in this paper. Point c is cut, and the detection profile is polished. The observation and identification are performed under the microscope. Four visual fields are selected and observed on the profile of each testing sample. Since the defect presents as dark in the images, Image-Pro (MediaCybernetics, Inc., Rockville, MD, USA) is used to mark the gray value of images. Thus, the void content is further calculated in terms of different gray values. Finally, the average void content of the four fields is utilized to assess the true void content at point c. This way, the relationship between the defect rate and the attenuation coefficient could be obtained.

**Figure 7:**
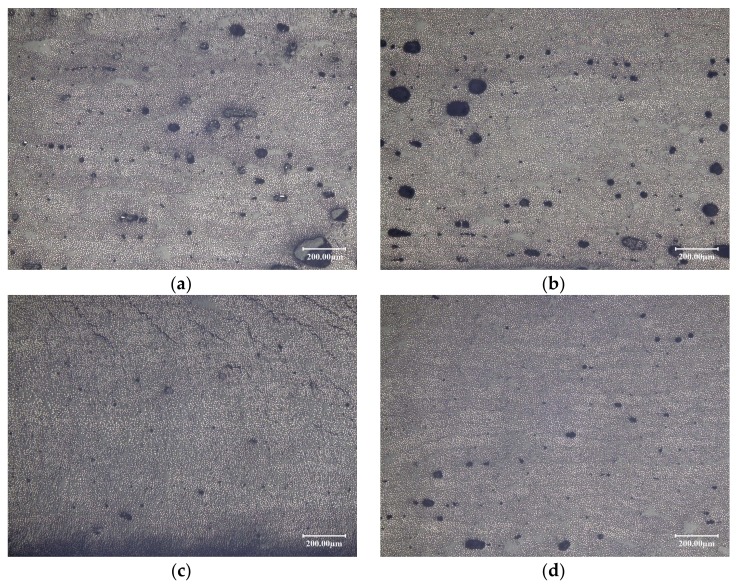
Manufacturing defects and identification of void content: (**a**) void content of 3.45% at *S* of 10 mm·s^−1^, *F* of 850 N, and *T* of 50 °C; (**b**) void content of 4.48% at *S* of 15 mm·s^−1^, *F* of 850 N, and *T* of 60 °C; (**c**) void content of 0.41% at *S* of 10 mm·s^−1^, *F* of 350 N, and *T* of 40 °C; and (**d**) void content of 0.82% at *S* of 15 mm·s^−1^, *F* of 600 N, and *T* of 50 °C.

The defects under the optical microscope are shown in Figure 7.

The calculation method of ultrasonic attenuation coefficient is shown in Equation (1):

(1) $$\alpha =\frac{20\log\frac{B_{1}}{B_{2}}}{x}$$

where α is the ultrasonic attenuation coefficient, *B*_1_ is the incident wave intensity of ultrasonic, *B*_2_ is the reflected wave intensity of the ultrasound, and *x* is the thickness of sample.

**Figure 8:**
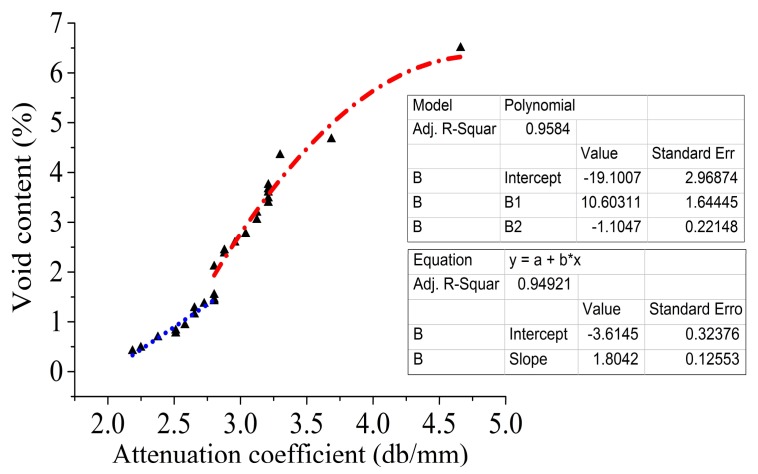
The relationship between the void content and the attenuation coefficient.

The relationship between the void content and the attenuation coefficient is shown in Figure 8.

In Figure 8, the relationship between the void content and the attenuation coefficient is divided into two parts. The void content is linearly related to the attenuation coefficient when the void content is less than 1.5%. The void content presents a quadratic nonlinear relationship with the attenuation coefficient when the void content is greater than 1.5%. When the void content is small, the attenuation of the ultrasound by the fibers and the resin contributes a large part of the total attenuation, which is approximately a linear relationship between the void content and attenuation. The attenuation effect of voids on ultrasound is gradually increased with the increase of the void content in contrast to the fibers and the resin, resulting in a nonlinear and rapid increase in ultrasonic attenuation. The linear fitting and polynomial fitting method are used to fit the curve of the relationship respectively, which is shown in Equation (2):

(2) $$p=\left\{ \begin{array}{ll} -3.61451+1.8042\alpha & p\leq 1.5\%\\ -19.10074+10.60311\alpha -1.1047\alpha^{2} & 1.5\%<p<7\% \end{array}\right.$$

where *p* is the void content.

When the void content is less than 0.5%, material properties have the greatest influence on the ultrasonic attenuation coefficient. When the void content is more than 0.5%, the contribution of the defect to the attenuation coefficient takes the dominant position [39]. On the other hand, the resin content is 33%, so the fiber is the main factor to affect the attenuation of ultrasonic absorption [40]. Therefore, although there is a certain error, it is feasible that the above model and the attenuation coefficient before curing are used to evaluate the void content before curing when the void content is large.

## 4. Results and Discussion

The experiment with the step of No. 12 is examined in this section in Figure 4.

### 4.1. Attenuation Model of Defects

**Figure 9:**
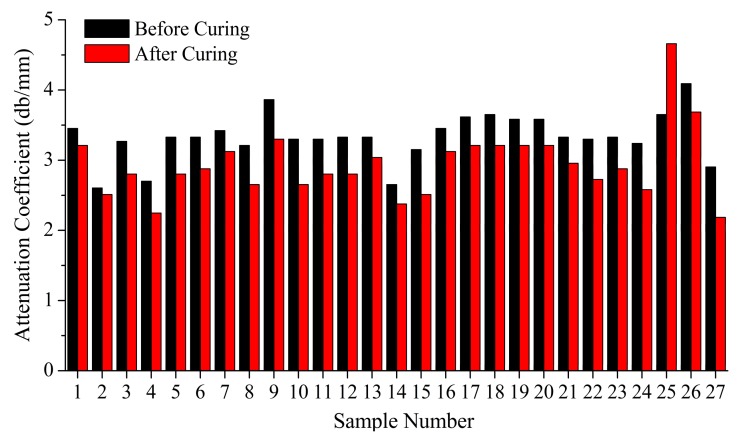
The average attenuation rates before and after curing.

By comparing the experimental results of 27 groups and the A-scan ultrasonic examination of 162 testing points before and after curing, the attenuation coefficient of each group are ranked. The results show that the attenuation coefficient of each group after curing can be consistent with that of each group before curing, basically. Further explanation shows that the defects in the laying process can be reduced after curing, but, relatively speaking, the defects produced during the laying process are still able to maintain this relative void content level after curing. The average attenuation rates before and after curing are shown in Figure 9.

**Figure 10:**
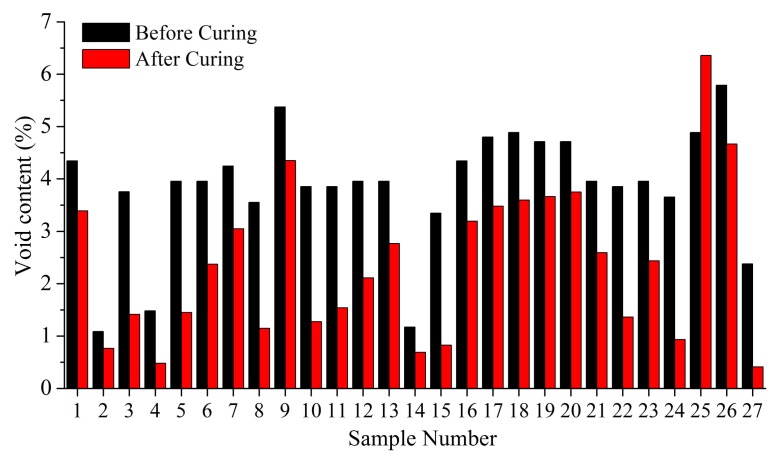
The average void content before and after curing.

The void content of the samples before curing is calculated through the relation model between the void content and the attenuation coefficient, which is shown in Figure 10.

### 4.2. Denoising Method

In order to compare the difference between the stress waveforms at different void content, the stress waves need to be filtered to remove the high-frequency vibration in the signals. At present, the main methods of the filtering transform are the Fourier transform and wavelet transform. A function will be expanded into different frequencies of the harmonic in the frequency domain using the Fourier transform. The original waveform is broken down into the linear superposition of sine wave or cosine wave. The Fourier transform is the spectral analysis of all the signals in the whole time domain. Hence, when the time domain changes into the frequency domain, the characteristics of the signals in the time domain are partly lost. Thus, there are some limitations for analysis of the signals that cannot guarantee relative stability with the time. The wavelet transform can be developed on the basis of the Fourier transform. The wavelet transform can preserve the characteristics of the time domain and frequency domain of the signals, which has certain adaptability to the characteristics of the signals. The filtering effect of the steady signal with some abrupt changes outperforms the Fourier transform. Therefore, wavelet theory is used to deal with the stress wave signal in this paper.

### 4.3. Laying Process

**Figure 11:**
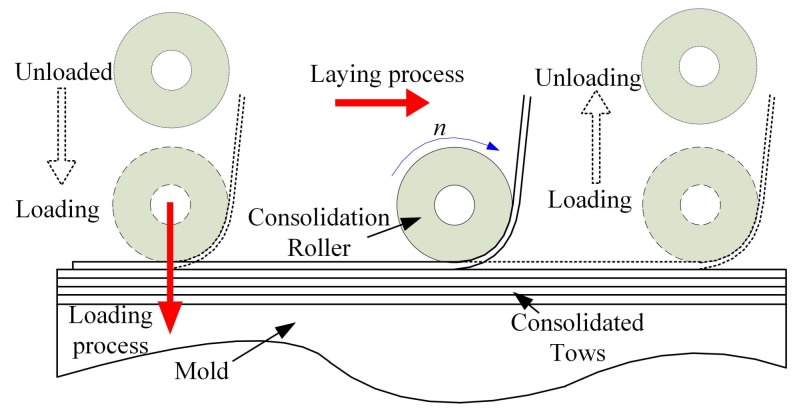
The laying process of the last tow.

This subsection aims at the analysis of the fourth part in the experimental scheme. On the basis of the existing 19 layers, where defects have formed and accumulated, the same processing parameters (laying speed of 20 mm·s^−1^, compaction force of 1.7 MPa) a pre-heating temperature of 40 °C is used to lay a tow with the sampling frequency of 8 kHz. The laying process of the tow is shown in Figure 11.

**Figure 12:**
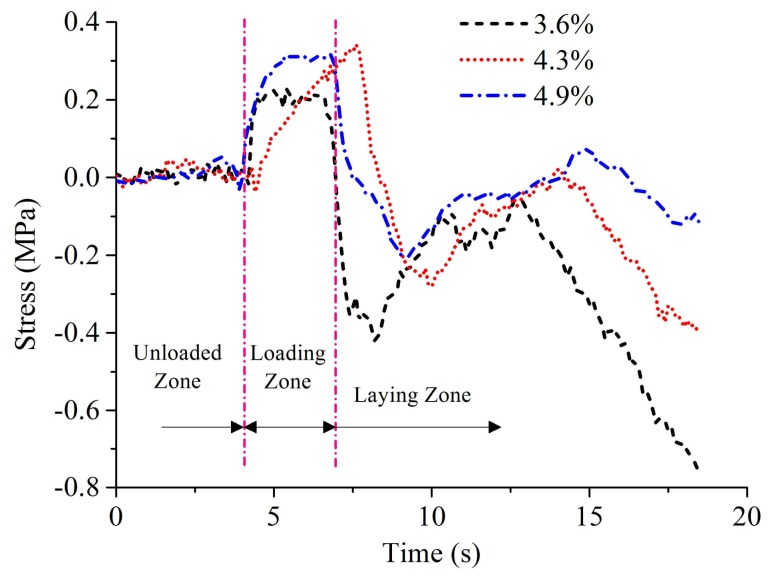
Stress waveforms at different void content during the laying process.

The complete laying process is divided into the following stages: The unloaded process refers to the fact that the consolidation roller has not touched the surface of the laminated plies during the dropping process of roller. The process from the laminated plies touched by the roller to the beginning of the laying process is called loading process. The last two stages are the laying process and unloading process. Therefore, the obtained stress waves also have such zones. The stress waveforms at different void content after four-level Haar wavelet transform are shown in Figure 12.

**Figure 13:**
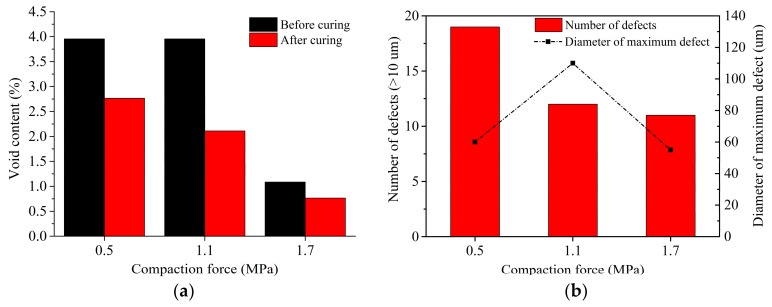
The relationship between defect distribution and compaction force at *S* of 20 mm·s^−1^ and *T* of 40 °C during the laying process: (**a**) void content; and (**b**) number of defects and diameter of maximum defect.

In terms of the calculation method of void content under different processing parameters, the relationship between defect distribution and compaction force is obtained, which includes void content, the number of defects, and the diameter of maximum defect. When other processing parameters remain consistent, the results show that most of the void content decreases with the increasing compaction force. Although the relationship between the diameter of the maximum defect and compaction force is not obvious, the number of large-diameter defects is closely related to the compaction force that shows negative correlation. The relationship between defect distribution and compaction force at *S* of 20 mm/s and *T* of 40 °C during the laying process is show in Figure 13.

**Figure 14:**
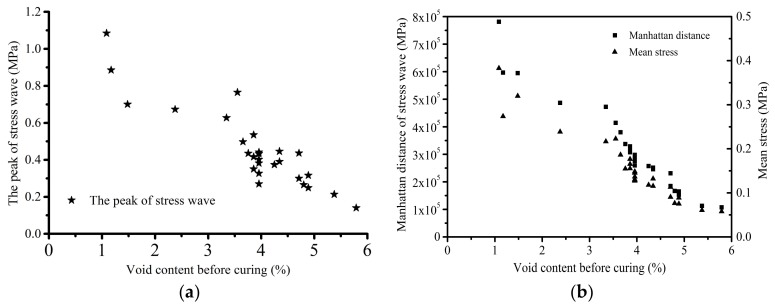
The relationship between the void content and the characteristics of stress wave during the laying process: (**a**) the peak; and (**b**) the Manhattan distance and mean stress.

In this section, the Manhattan distance, peak value, and mean stress value of the stress waves are taken as the characteristic variables to analyze the effect of different void content on the stress waves. The Manhattan distance of the stress wave refers to the sum of the absolute values of each test stress. In Figure 14, the relationship between the void content and the Manhattan distance, peak, and mean stress is displayed respectively during the laying process. It is found that the void content has a nonlinear negative correlation with the peak, Manhattan distance, and mean stress. Due to the stress concentration and wave scattering around the defects, the components with higher void content could lose more energy in the process of stress wave propagation. Therefore, their peak, Manhattan distance, and mean stress are lower than that of the components with lower void content.

## 5. Conclusions

In this paper, the relationship between the stress wave and the initial internal defect is established by analyzing the propagation characteristics of the stress wave under different defect rates. The following conclusions are obtained:

(1) When the defect rate is less than 1.5%, the defect rate is linearly positively related to the attenuation coefficient. When the defect rate is more than 1.5%, the defect rate presents a quadratic nonlinear positive correlation with the attenuation coefficient.
(2) The curing process can effectively reduce the defects in the laying process. According to the ranking of the defect rate with Twenty-seven groups before and after curing, it indicates that higher defect rates produced by the laying process are still able to maintain the relatively higher ranking of defect rates after curing.
(3) Due to the scattering of energy and the stress concentration around the defects during the laying process, the higher the defect rate is, the greater the potential energy losses. Thus, there is a non-linear negative correlation between the defect rate and the characteristics of stress waves, including the peak, Manhattan distance, and mean stress during the laying process.

Future work includes further improving the accuracy of wave velocity measurement, and attempting to establish an online real-time detection and control system for internal defects based on stress wave characteristics.

## Figures and Tables

**Table 1:** Some typical properties of test prepreg.

Properties	Fiber volume content	Fiber weight	Prepreg resin content	Prepreg areal weight
Value	65%	150 g·m^−2^	33%	224 g·m^−2^

**Table 2:** Experimental parameters.

Level	1	2	3
Laying speed	10 mm·s^−1^	15 mm·s^−1^	20 mm·s^−1^
Compaction force	0.5 MPa (350 N)	1.1 MPa (600 N)	1.7 Mpa (850 N)
Pre-heating temperature	40 °C	50 °C	60 °C
